# Circulating and intrarenal renin–angiotensin systems in healthy men and nonpregnant women

**DOI:** 10.14814/phy2.12586

**Published:** 2015-10-14

**Authors:** Kirsty G Pringle, Shane D Sykes, Eugenie R Lumbers

**Affiliations:** 1 School of Biomedical Sciences and Pharmacy, University of NewcastleNewcastle, New South Wales, Australia; 2 Mothers and Babies Research Centre, Hunter Medical Research InstituteNewcastle, New South Wales, Australia

**Keywords:** Circulating, intrarenal, renin–angiotensin system, urinary

## Abstract

The urinary excretion of renin–angiotensin system (RAS) proteins could reflect the activity of the intrarenal RAS. We hypothesized that the rates of excretion of RAS components into human urine are independent of circulating levels of these proteins and reflect the intrarenal RAS. There are no reports of the simultaneous measurement of prorenin, active renin, angiotensinogen (AGT), and angiotensin-converting enzyme (ACE) excretion in healthy individuals. Therefore, we measured plasma prorenin, ACE, and AGT and urinary renin (uRenin), prorenin (uProrenin), ACE (uACE), and AGT (uAGT) in men and nonpregnant women. Plasma (p) AGT was higher in women then men. Women who were taking estrogen had significantly higher pAGT. In women, pProrenin was negatively correlated with pAGT. There were no correlations between pProrenin, pAGT, and pACE and their urinary counterparts in either men or women. In men, uProrenin/creatinine ratios were lower than in women. There was no effect of estrogen use on urinary excretion of pProrenin, renin, AGT, and ACE. In men, there were significant correlations between uACE/creat and uRen/creat and uAGT/creat; uProrenin/creat and plasma cystatin C levels; and uRenin/creat and uNa/K were also positively correlated. No associations were found in women. In conclusion, urinary excretion of prorenin is sexually dimorphic and is not affected by estrogen use in women. Our data also suggest that the relationship between renal handling of sodium and urinary renin is sexually dimorphic. Since we found no associations between plasma RAS proteins and their urinary counterparts, and the ratio of uProrenin:pProrenin was strikingly different between men and women, levels of urinary RAS proteins in individuals with normal kidney function are most likely the result of tubular secretion, rather than ultrafiltration.

## Introduction

There is growing interest in the urinary excretion of renin–angiotensin system (RAS) proteins because it is claimed that urinary angiotensinogen (uAGT), in particular, reflects the activity of the intrarenal RAS. uAGT levels are elevated in patients with chronic kidney disease (CKD) and diabetic nephropathy (Yamamoto et al. 2007; Kobori et al. 2008; Urushihara et al. 2010; van den Heuvel et al. 2011; Kobori and Navar 2011; Nishiyama et al. 2011; Roksnoer et al. 2013) and are reduced when patients are treated with RAS blocking drugs (Kobori et al. 2009; Urushihara et al. 2010). The presence of angiotensin-converting enzyme (ACE) (Kokubu et al. 1978; Ura et al. 1985), renin (Lumbers and Skinner 1969a,b; Haley and Johnson 1978), and AGT (Lumbers and Skinner 1969a; Favaro et al. 1972) in urine has previously been described, however there are very few studies comparing their excretion rates within the same individual even though all these components are distributed along the renal tubule (Lumbers and Skinner 1969a; Darby and Sernia 1995; Metzger et al. 1999; Rohrwasser et al. 1999).

Intrarenal generation of Ang II and subsequent binding of angiotensin II (Ang II) to the Ang II type 1 receptor (AT_1_R) causes direct renal vasoconstriction, alters sensitivity of the tubuloglomerular feedback mechanism and stimulates proximal and distal tubular sodium reabsorption (Braam et al. 1993; Navar 1997). This tubular Ang II/AT_1_R interaction has been shown to be responsible for the maintenance of hypertension in mice chronically infused with Ang II (Crowley et al. 2005, 2006).

The urinary excretion of AGT has been most extensively studied, particularly in patients with proteinuria. It has been claimed that urinary excretion of AGT occurs with proteinuria because it can escape through the damaged filtration barrier from plasma (van den Heuvel et al. 2011; Roksnoer et al. 2013). This could account for the positive correlations seen between uAGT and urinary albumin in proteinuria (van den Heuvel et al. 2011; Roksnoer et al. 2013). Furthermore, in transgenic mice renal AGT and intrarenal generation of Ang II are diminished when hepatic AGT is “knocked out” but not when renal AGT is specifically knocked out (Matsusaka et al. 2012). Intrarenal AGT and Ang II levels are further increased when the filtration barrier is selectively disrupted due to podocyte damage; supporting the concept that uAGT/creatinine is elevated when the filtration barrier is damaged (Matsusaka et al. 2012). Saito and colleagues have demonstrated that uAGT/creatinine ratios are increased in patients with diabetic nephropathy before they develop microalbuminuria (Saito et al. 2009). Furthermore, in animals made diabetic with streptozotocin, uAGT is consistently elevated prior to the onset of gross albuminuria (Kamiyama et al. 2012). Therefore, there is a dispute as to whether uAGT/creatinine is a marker of increased activity of the intrarenal RAS or whether it is present in the urine because the barrier is damaged. We propose that in healthy individuals, the levels of urinary RAS components reflect the intrarenal RAS rather than ultrafiltration of the circulating RAS components.

In the current study, we measured uProrenin, uRenin, uACE, and uAGT levels in urine from healthy men and nonpregnant women without proteinuria to see if there were any differences between the two sexes in the excretion of RAS proteins. Differences in plasma (p) RAS proteins, including prorenin (Derkx et al. 1986; Danser et al. 1998) and AGT (Skinner et al. 1969; Clauser et al. 1989) between men and women, have been documented but the effect of sex has not been considered when the urinary excretion of RAS proteins has been studied. This is despite the fact that Lumbers and Skinner showed that the renal clearance of total renin (active renin and prorenin) was greater in women than men (Lumbers and Skinner 1969a). Finally, we determined the interrelationships between pRAS and uRAS components and the relationships between them and the excretion of albumin or protein.

## Materials and Methods

### Study design

Men (*n *=* *10) and nonpregnant women (*n *=* *10) were recruited. Informed consent was obtained from each participant and information such as height and weight, age, current medical conditions, and medications were recorded. None of the participants had any significant medical condition. The mean age for men was 36.2 years (range: 21–57) and for women was 28.2 years (range: 19–48). Four of the women were using estrogen-containing medications, three women were using oral contraceptives (Yasmin, Diane 35 and Levlen ED), and one was using a hormone replacement cream (Biest Cream, 0.5% Progesterone 2% UNG PCCA).

Ethical approval for this work was obtained from the Hunter New England Human Research Ethics Committee (HNEHREC 08/05/21/4.01) and the University of Newcastle Human Research Ethics Committee (H-2009-0177).

### Sample collection

Blood was collected by venipuncture into EDTA vacutainers and handled at room temperature until centrifugation. Blood for biochemical analyses was collected into lithium–heparin tubes and placed on ice until centrifugation at 4°C. Samples were processed by Hunter Area Pathology Service, Newcastle, NSW. Blood samples were spun at 3000 *g* for 15 min. EDTA blood was spun for 15 min at 1500 *g*. Urine samples were collected by each participant and kept at room temperature prior to storage at −20°C.

### Urine dialysis and concentration

Urine samples were dialyzed to pH 7.5 in 20 kDa Slide-A-Lyzer dialysis cassettes (Thermo Fisher Scientific, IL), which had been presoaked in phosphate–phosphate buffer (0.012 mol/L NaH_2_PO_4_.2H_2_O, 0.087 mol/L Na_2_HPO_4_.12H_2_O, 0.001 mol/L EDTA, 0.075 mol/L NaCl, pH 7.5) and weighed before sample addition. Samples were dialyzed against this pH 7.5 phosphate–phosphate buffer for 24 h at room temperature with constant stirring and one change of buffer. Cassettes were weighed after completing dialysis and samples restored to their original volume by addition of distilled H_2_O. Aliquots of dialyzed urine were then removed and stored at −20°C for measurement of uACE and uAGT.

Samples were then concentrated for measurement of prorenin and active renin. Dialyzed urine was placed in Amicon Ultra-15, 10 kDa centrifugal filters (Merck Millipore, Darmstadt, Germany) and concentrated ∼5× by centrifugation at 4000 *rcf* at room temperature. The exact volume of each sample was recorded after concentration so that the degree to which they were concentrated could be calculated. Concentrated urine samples were stored at −20°C for the subsequent measurement of active renin and prorenin.

### Laboratory measurements

Urinary and plasma creatinine, albumin, and protein were measured using Siemens Flex reagent cartridges for the respective proteins, while electrolytes were measured using a Siemens V-Lyte integrated multisensor and all read using a Siemens Dimension Vista 1500 chemistry analyzer at Hunter Area Pathology Service (Newcastle, NSW, Australia).

Plasma cystatin C was measured at Pathology New England (Tamworth, NSW, Australia) using a Gentian Cystatin C Immunoassay read on a Beckman Coulter DxC 600 analyzer.

Commercially available enzyme-linked immunosorbent assay (ELISA) kits were used to measure plasma and urinary prorenin (Molecular Innovations, MI), ACE (Duoset, R&D systems, MN), and AGT (IBL International, Hamburg, Germany) according to the manufacturer’s instructions (see also [Sykes et al. 2014a,b]). The intra- and interassay coefficient of variation (CV) for each assay was 26.5% and 16.9% (*n *=* *2 plates) for ACE, 7.7% and 7.9% (*n *=* *2 plates) for AGT, respectively. The intra-assay CV for the prorenin assay was 17.1%. The lowest values we could successfully measure for uACE, uAGT, and uProrenin were 0.001 ng/mL, 0.2 ng/mL, and 0.6 pg/mL, respectively.

### Urinary active renin measurement

Active renin was measured using methods modified from techniques used to measure plasma/amniotic fluid renin in the presence of excess substrate from nephrectomized sheep plasma (NSP) (Lumbers and Lewes 1979; Marsh et al. 2001; Gibson et al. 2006). Dialyzed, concentrated urine was added to an equal volume of NSP, 1.4 mmol/L phenylmethylsulfonylfluoride (PMSF), and additional phosphate–phosphate buffer (pH 7.5) to bring samples to a final volume of 1.1 mL. Samples were vortexed and pulse spun in a centrifuge prior to incubation at 37°C for 24 h. At the end of the incubation, samples were snap-frozen and sent to the local pathology service. The amount of Ang I generated was measured using a Diasorin GammaCoat Plasma Renin Activity ^125^I RIA kit (Diasorin, MN). Urinary active renin levels were expressed as the rate at which Ang I was generated (ng/mL/h) from NSP at pH 7.5 and 37°C. The intra-assay CV for uRenin measurement was 16% (*n *=* *3). Plasma active renin was not measured because it is sensitive to acute changes in posture and diet.

### Data analyses

Mann–Whitney *U* tests were used to identify significant differences between men and women. Spearman’s correlations were conducted using matrices and pairwise comparisons to verify whether relationships of interest were statistically significant. Statistical significance was determined as *P *<* *0.05. Data were analyzed using Stata/IC 11.0 (StataCorp LP, TX) and GraphPad Prism 6.0 (GraphPad Software, Inc., CA) and are reported as median and interquartile range (IQR).

## Results

**Figure 1:**
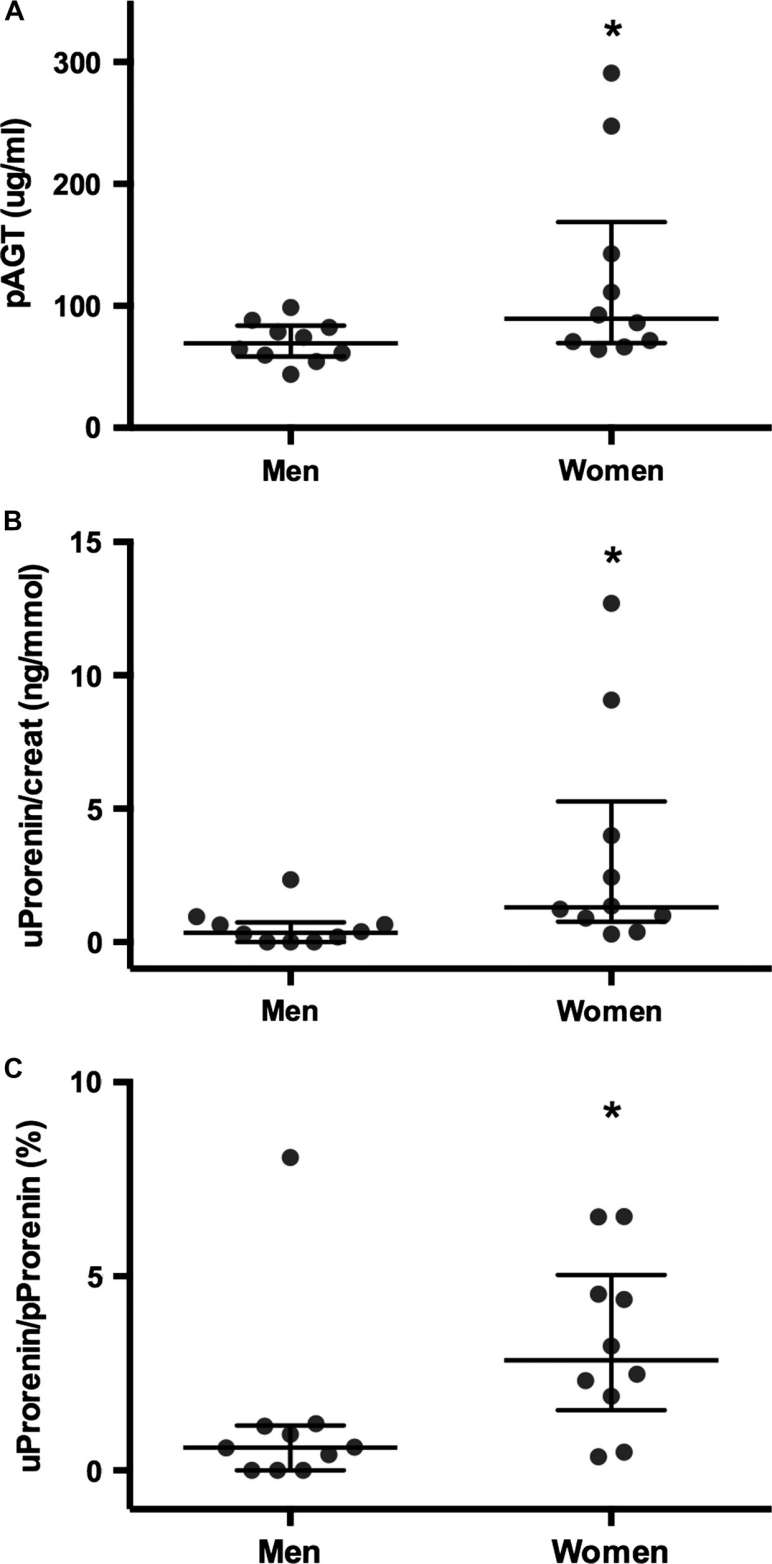
Concentrations of (A) plasma AGT (pAGT) and (B) urinary prorenin/creatinine (uProrenin/creat) in healthy men and women. (C) The levels of prorenin in urine (uProrenin) expressed as a percent of that found in plasma (pProrenin) were significantly higher in women than men. *P <* *0.05. Data are presented as individual values with median and interquartile ranges indicated.

Plasma AGT levels were significantly higher in women (median: 89.4 *µ*g/mL, IQR: 69.6–168.9) than in men (69.4 *µ*g/mL, IQR: 58.4–83.9, *P *<* *0.05, Fig.1A). A similar sex difference was observed in uProrenin/creat ratios, which were also significantly higher in women (median: 1.3 ng/mmol, IQR: 0.77–5.27) compared to men (median: 0.35 ng/mmol, IQR: 0.0008–0.74, *P *=* *0.01, Fig.1B). In women, the levels of uProrenin were 2.47% of that found in plasma, which was significantly higher (*P* = 0.028) than the value obtained in men for uProrenin as a % of pProrenin (0.67%, Fig.1C). These were the only sex differences identified; values for other variables are shown in Table1.

**Table 1:** Plasma and urinary excretion of RAS proteins as well as the plasma creatinine and cystatin C, and urinary protein-to-creatinine, and albumin-to-creatinine ratios measured in men and nonpregnant women

Variables	Men		Women	
	Median (IQR)	*n*	Median (IQR)	*n*
Plasma				
Prorenin (pg/mL)	523 (429–1263)	10	322 (203–616)	10
ACE (ng/mL)	192.4 (165.3–236.4)	10	180.6 (135.8–222.1)	10
Creatinine (umol/L)	75 (72–78)	5	72 (66–76)	4
Cystatin C (mg/L)	0.64 (0.61–0.66)	10	0.65 (0.53–0.69)	10
Urine				
ACE/creat (*μ*g/mmol)	0.011 (0.005–0.018)	10	0.019 (0.004–0.041)	10
AGT/creat (*μ*g/mmol)	0.863 (0.335–1.402)	10	1.200 (0.374–1.600)	10
Renin/creat (*μ*g/h/mmol)	0.013 (0.004–0.023)	10	0.024 (0.014–0.036)	10
Protein/creat (mg/mmol)	2.6 (1.8–8.3)	10	2.5 (1.9–6.3)	10
Albumin/creat (mg/mmol)	1.6 (0.8–4.0)	10	1.1 (0.8–3.0)	10

Creat, creatinine; IQR, interquartile range.

**Figure 2:**
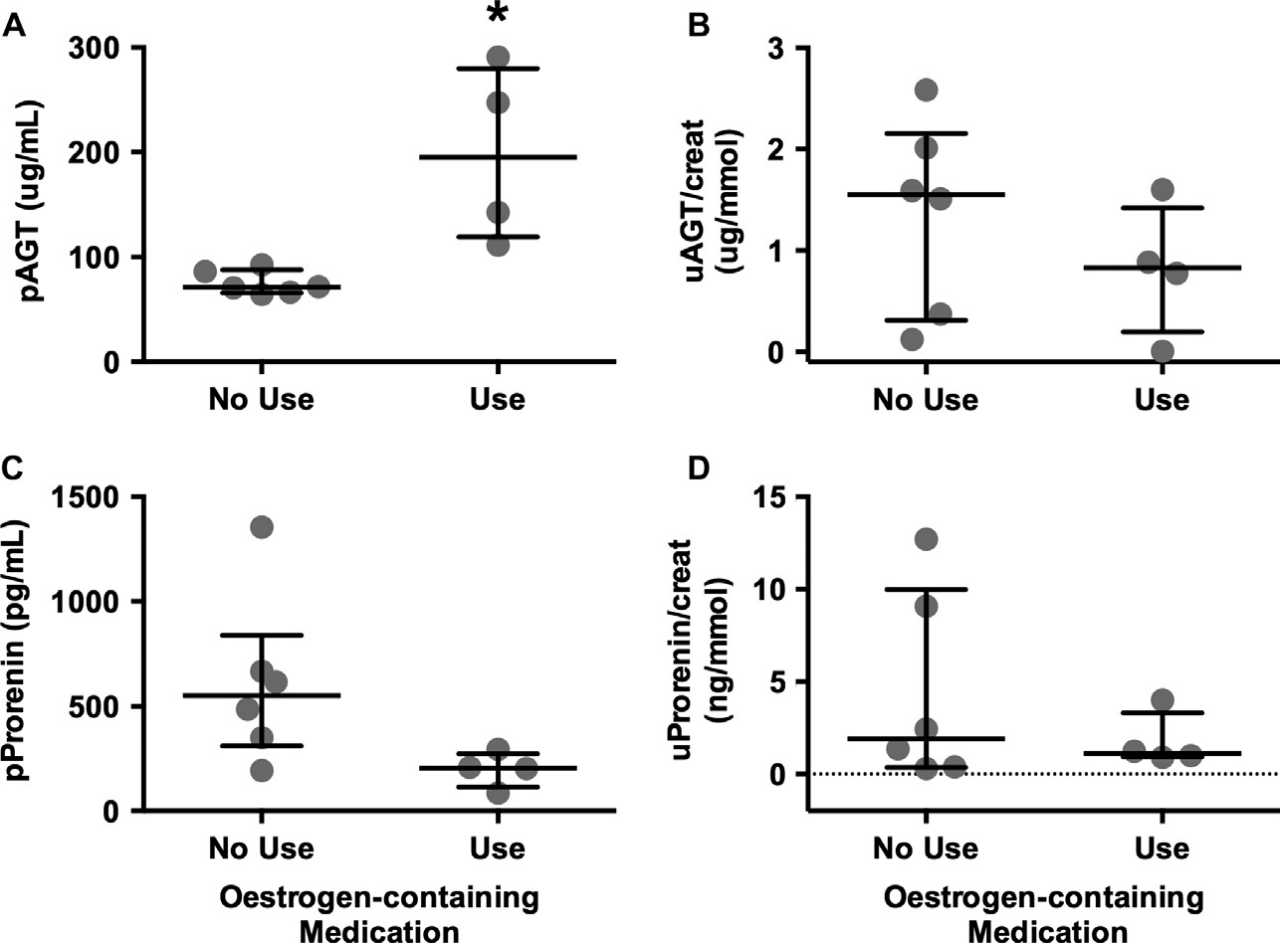
The effect of estrogens on (A) plasma AGT, (B) uAGT/creat, (C) plasma prorenin, and (D) uProrenin/creat ratios in women are divided according to whether they were (*n *=* *4) or were not taking estrogens (*n *=* *6). *P <* *0.01. Data are presented as individual values with median and interquartile ranges indicated.

It should be noted that pAGT was higher in women taking estrogens (*n *=* *4) compared with women who were not (*n *=* *6, *P *<* *0.01, Fig.2). In those women not taking estrogens, pAGT levels were similar to those levels in men (*P *=* *0.5). Conversely, plasma prorenin levels in women taking estrogens tended to be lower than levels in women not using estrogens (*P *=* *0.07, Fig.2). No differences were seen in uAGT/creat or uProrenin/creat ratios between women taking estrogens and those who were not (Fig.2).

There were no differences in plasma creatinine, plasma cystatin C (a surrogate measure of GFR), urinary protein/creatinine, or urinary albumin/creatinine between men and women (Table1). It is important to note, however, that 4/10 men and 2/10 women had urinary samples with albumin/creatinine ratios above the normal range (>2.4 and >3.4 mg/mmol and for men and women, respectively).

### Interrelationships between pRAS and uRAS components

pProrenin levels were positively correlated with pACE in men (*ρ* = 0.685, *P *<* *0.03, Table2). In women, pProrenin levels were negatively correlated with pAGT levels (*ρ* = −0.697, *P *<* *0.03, Table2).

**Table 2:** Correlations between plasma and urinary RAS in men and nonpregnant women

Variables	Men		Women	
	*ρ*	*P*	*ρ*	*P*
Plasma RAS				
pProrenin				
pACE	**0.685**	**0.03**	0.491	0.155
pAGT	−0.370	0.707	**−0.697**	**0.03**
Urinary RAS				
uACE/creat				
uRenin/creat	**0.673**	**0.03**	0.515	0.133
uAGT/creat	**0.649**	**0.04**	0.467	0.179

Data were analyzed within each group using Spearman’s correlations to verify whether relationships of interest were statistically significant. Statistically significant correlations are in bold font. *ρ*, Spearman’s rho; creat, urinary creatinine; u, urinary; p, plasma.

Only in men were there any interrelationships between uRAS proteins. In men, there were significant correlations between uACE and uRenin (*ρ* = 0.673, *P *=* *0.03) and between uACE and uAGT (*ρ* = 0.649, *P *=* *0.04, Table2).

There were no associations between pRAS proteins and their corresponding uRAS proteins (data not shown). In men, however, a negative correlation between pACE and uProrenin/creat ratios was found (*ρ* = −0.736, *P *<* *0.02).

### Relationships between plasma and urinary RAS proteins and renal function

In women, pACE and uNa/K ratios were directly correlated (*P *=* *0.04, Table3), while pAGT levels were negatively correlated to urinary albumin and protein/creat ratios (*P *=* *0.04, *P *<* *0.02, respectively). There were no correlations between pRAS proteins and renal function in men.

**Table 3:** Correlations between the plasma RAS, urinary RAS, and markers of renal function in men and nonpregnant women

Variables	Men		Women	
	*ρ*	*P*	*ρ*	*P*
Plasma RAS				
pACE				
uNa/K	0.115	0.759	**0.649**	**0.043**
pAGT				
pCysC	0.086	0.815	0.280	0.431
uAlbumin/creat	−0.511	0.129	**−0.651**	**0.042**
uProtein/creat	−0.438	0.199	**−0.721**	**0.019**
Urinary RAS				
uAGT/creat				
uAlbumin/creat	0.322	0.361	0.073	0.845
uProrenin/creat				
pCysC	**0.721**	**0.019**	−0.073	0.830
uProtein/creat	0.159	0.659	0.467	0.179
uRenin/creat				
uAlbumin/creat	−0.036	0.911	−0.024	0.938
uProtein/creat	0.219	0.542	0.127	0.733
uNa/K	**0.77**	**0.009**	−0.309	0.387

Data were analyzed within each group using Spearman’s correlations to verify whether relationships of interest were statistically significant. Statistically significant correlations are in bold font. *ρ*, Spearman’s rho; creat, urinary creatinine; u, urinary; p, plasma; Na/K, sodium-to-potassium ratio; CysC, Cystatin C.

In men, plasma cystatin C levels and uProrenin/creat ratios were positively correlated (*P *<* *0.02, Table3); uRenin/creat ratios and uNa/K ratios were also correlated (*P *<* *0.01). There were no associations between the excretion of uRAS proteins and renal function in women.

## Discussion

This is the first study that compares, in healthy men and women, the simultaneous excretion of urinary RAS proteins and examines their interrelationships as well as their relationships with indices of renal function and their association with plasma levels of the same proteins.

It has been previously reported that men have higher plasma prorenin levels than women (Danser et al. 1998; van den Heuvel et al. 2011). In the current study, this difference did not reach statistical significance, even though almost half of the women were taking an estrogen-containing medication, which tended to lower plasma prorenin levels (Fig.2). Women who were taking estrogen-containing medications had higher pAGT levels (Fig.2) because AGT has an estrogen-sensitive response element situated between the TATA box and the start of the transcription site (Derkx et al. 1986; Yanai et al. 1996; van den Heuvel et al. 2011). The high pAGT presumably acts via a negative feedback pathway to inhibit renin and prorenin release from the kidney, as has been found in other studies where both prorenin and active renin levels are lower in women taking oral contraceptives (Derkx et al. 1986; Danser et al. 1998). Interestingly the women in our study did not have higher uAGT levels, even though it has been shown that kidney AGT, like hepatic AGT, is responsive to estrogens (Klett et al. 1993).

Since prorenin and active renin both required the concentration of urine samples prior to measurement, this made the measure of these uRAS proteins more difficult. Furthermore, even with this concentration step, uProrenin was often not detected. Of all the assays conducted, the AGT ELISA had the lowest inter- and intra-assay CVs and could be measured reliably in unconcentrated urine. Thus, uAGT was the easiest and most sensitive uRAS protein measured.

In the current study, we found no relationship between pAGT and uAGT/creat, or between any measured pRAS protein and its urinary counterpart. In these healthy subjects it would also seem that the excretion of uRAS proteins is independent of glomerular filtration. We have come to this conclusion for the following reasons:

1. The finding that uProrenin was a much greater percent of pProrenin in women compared with men makes it unlikely that the presence of this protein in urine is simply the result of ultrafiltration.
2. There was no change in uAGT in women taking estrogens who had elevated pAGT levels (Fig.2).
3. In women, there were negative correlations between pAGT and urinary albumin and protein/creatinine ratios (Table3). This correlation would not have occurred if AGT in the urine was solely the result of ultrafiltration.
4. In men, there were direct relationships between uACE and uRenin and between uACE and uAGT. ACE is an ectoenzyme situated along the renal tubular brush border (170 kDa [Soubrier et al. 1988]); the association between its excretion and the excretion of two molecules of very different size (renin, 48 kDa and AGT, 65 kDa, [van den Heuvel et al. 2011]) means it is unlikely that they are in urine as a result of glomerular ultrafiltration. They most likely reflect activity of an intratubular RAS.

Lumbers and Skinner (1969a) have previously shown that renin clearance by the kidney is greater in women than in men. However, they used an assay which depended on measuring Ang I generation from nephrectomized sheep plasma at pH 7.5 and 37°C after samples had been “acid activated” by dialysis against a pH 3.3 containing buffer (Lumbers and Skinner 1969a; Lumbers 1971; Morris and Lumbers 1972). Thus, it is interesting that we showed that uProrenin/creat ratios were greater in women than in men using a direct method for measuring prorenin protein (Fig.1). A study by van den Heuvel et al. (2011) had previously demonstrated that there was no sexual dimorphism in urinary components of the rennin–angiotensin aldosterone system (RAAS), however, urinary prorenin was reported to be undetectable in their study, which included both diabetic and nondiabetic subjects.

We found no differences in uRenin/creatinine ratios between men and women. Thus, the differences in renal clearance of total renin reported previously Lumbers and Skinner (1969a) were probably due to the greater clearance of prorenin by women compared with men. It is also interesting to note that while Lumbers and Skinner showed no relationship between total renin excretion and sodium output (Lumbers and Skinner 1969a), in the current study we found a direct correlation between uRenin/creat and uNa/K ratios in men but not between urinary prorenin/creat and uNa/K. If uRenin/creat reflects the activity of the intratubular RAS, it is possible that it is influenced by the intratubular sodium concentration reaching the distal nephron.

Although we did not see any positive correlation between uAGT/creat and uAlbumin/creat in this cohort of healthy individuals, a positive relationship between urinary albumin and uAGT/creat has been reported on numerous occasions in pathological settings (Kobori et al. 2009; van den Heuvel et al. 2011; Sawaguchi et al. 2012). A previous study examining a cohort of hypertensive patients with or without diabetes mellitus also found a significant correlation between the plasma/urinary ratios for albumin and AGT, but no correlation between the plasma/urinary ratios for renin and albumin (van den Heuvel et al. 2011).

In conclusion, we have measured the urinary excretion of active renin, prorenin, ACE, and AGT in a cohort of healthy men and women and shown that their excretion shows sexual dimorphism, that is, uProrenin/creat ratios were lower in men. We also detected no correlations between pRAS and uRAS components suggesting that the circulating and intrarenal rennin–angiotensin systems are acting independently in the absence of any pathology.
